# Function of weaponry in females: the use of horns in intrasexual competition for resources in female Soay sheep

**DOI:** 10.1098/rsbl.2007.0278

**Published:** 2007-08-21

**Authors:** Matthew R Robinson, Loeske E.B Kruuk

**Affiliations:** Institute of Evolutionary Biology, School of Biological Sciences, University of EdinburghWest Mains Road, Edinburgh EH9 3JT, UK

**Keywords:** female aggression, intrasexual competition, polymorphism, sexual selection

## Abstract

In many species, females show reduced expression of a trait that is under sexual selection in males, and this expression is thought to be maintained through genetic associations with the male phenotype. However, there is also the potential for the female trait to convey an advantage in intrasexual conflicts over resources. We tested this hypothesis in a feral population of Soay sheep, in which males and females have a polymorphism for horn development, producing either full (normal horned), reduced (scurred) or no (polled, females only) horns. During the lambing period, females who possessed horns were more likely to initiate and win aggressive interactions, independent of age, weight and birthing status. The occurrence of aggression was also context dependent, decreasing over the lambing period and associated with local density. Our results demonstrate that a trait that confers benefits to males during intrasexual competition for mates may also be used by females in intrasexual competition over resources: males use weaponry to gain mates, whereas females use weaponry to gain food.

## 1. Introduction

In polygynous mammals, intrasexual aggression typically differs between the sexes, with males coming into conflict over breeding opportunities while females come into conflict over resources such as food and space (Thouless & Guinness 1986; Festa-Bianchet 1991). Aggressive competition for reproductive opportunities in males may lead to the evolution of traits such as horns or antlers, which are associated with breeding success (Andersson 1994). In many species, females show reduced expression of a male sexually selected trait. Darwin (1874) explained this through a process of ‘inheritance’ and Lande (1980) demonstrated that despite costs of displaying the trait, female expression may be maintained through genetic associations between the sexes. An alternative hypothesis exists where females benefit from the expression of secondary traits, with trait size as a signal of condition which females use to assess each other at distance (West-Eberhard 1983; Amundsen 2000). However, previous studies have found that horns and antlers do not directly influence the outcome of female intrasexual interactions, particularly when age is taken into account (e.g. Barrette & Vandal 1986; Holand *et al.* 2004). If female horns have a direct function in intrasexual conflict the possession of the trait would convey a direct advantage in competition over resources. Therefore, the potential would exist for the trait to be maintained within the population as a result of positive selection.

To assess the function of female horns, we examine the relationship between female intrasexual aggression and horn type in a free-living population of Soay sheep (*Ovis aries*) on the Island of Hirta, St Kilda, Scotland. Soay sheep display a polymorphism for horn development, with the two sexes producing either a full horn (normal horned), a reduced horn (scurred) or no horns (polled, females only). Therefore, they provide an ideal opportunity to examine the relationship between aggressive interactions and secondary trait development in females, independent of body size and age. Furthermore, low grass growth and high female resource demand, during early spring, mean that requirements for resources may exceed their availability (Clutton-Brock & Pemberton 2004). Therefore, we also examine female intrasexual aggression in relation to breeding time and density of resource use.

## 2. Material and methods

### (a) Study system

The present study focuses on an unmanaged, feral population of Soay sheep (*O. aries*) residing on the island of Hirta within the St Kilda archipelago in the North Atlantic (57°49′ N, 08°34′ W). Since 1985, individuals within the Village Bay study population have been intensively monitored throughout their life, with 95% of lambs born being ear tagged (for a detailed description, see Clutton-Brock & Pemberton (2004)). Behavioural observations on aggression have not previously been conducted upon the females of this population.

### (b) Behaviour

During a single lambing period (April–May 2006), we conducted fifty, 1 h sampling periods, divided between three areas of similar fixed size. First, we recorded the identities of all females present within each area, determining local density and the reproductive status (whether they had given birth or not) of each female observed. As individuals have been followed from birth, recording their identity allowed us to determine the age and horn type of every individual in the sample. Second, all-occurrence sampling (Altmann 1974) was conducted for 1 hour within each area, recording aggressive interactions between females. Aggressive behaviour was defined as displacement of another individual through movement (move or turn towards), body threats (ears back or legs forward) or head butting. The conflict was considered to be resolved when an individual withdrew from the area in which they were feeding. A winner and a loser were identified for every interaction, and in all aggressive interactions the winner was the initiator. Three observers rotated between areas and as there was no evidence of any effect of area or observer we excluded these factors from our analyses. Censuses recorded the identity of 185 females, a total of 862 times and over the 50 independent sampling periods we recorded 51 aggressive interactions involving 39 different individuals. Soay sheep forage in predominantly single-sex herds (Clutton-Brock & Pemberton 2004) and thus we observed only intrasexual interactions.

### (c) Morphometric measurements

Morphometric measures are recorded in a two-week period in August each year when approximately 65% of the study population are caught and measured. We included measures recorded in August 2005 of the horn length of normal-horned females (measures were available from 40 individuals, which were recorded 165 times), individual body weight (in kilogram) and hindleg length (distance between tubercalcis of fibular tarsal bone to the distal end of the metatarsus: a measure of body size). Body weight and hindleg measures were available for 75 females upon which 315 observations were made.

### (d) Statistical analysis

We divided our statistical analyses into two parts. In the first analysis, we conducted a generalized linear mixed model (GLMM) with binomial error structure to determine factors associated with female aggression. All of the individuals observed in each census were recorded as aggressive (1, i.e. initiating aggression) or non-aggressive (0) in the following sampling period. Fixed effects included age, horn type, reproductive status, the density of females in the area under observation (local density) and the proportion of those with lambs. The model was reduced in a stepwise manner and only the final model is shown. Including morphometric measures reduced our sample sizes; therefore, we tested for their effects by adding them to the final model.

Second, we conducted a GLMM with binomial error structure based upon the observed aggressive interactions to test for differences in age and horn phenotype between the winner and loser of each interaction. A focal individual was selected at random from each interacting pair and classified as either the winner (1) or loser (0). Relative age (age difference between the focal and the second individual) was included as a linear fixed effect, alongside relative horn type (difference between the focal and the second individual in ranked horn phenotype: polled=0; scurred=1; normal horned=2). Including both fixed effects within one model separated the effects of age and weaponry.

In both GLMMs, the fixed effect significance was assessed using Wald statistics tested against a *Χ*^2^ distribution on the appropriate degrees of freedom. Including individual as a random effect accounted for repeated observations on individuals. Analyses were conducted using Genstat (Lawes Agricultural Trust 2007).

## 3. Results

### (a) Occurrence of aggression

The initiation of female intrasexual aggressive interactions was associated with an individual's horn type, age and reproductive status (table 1). Normal-horned females were more likely to be aggressive than scurred or polled females (figure 1*a*). Older individuals within a group were also more likely to be aggressive (figure 1*b*), with no evidence of an interaction between age and horn type (Wald_1_=0.18, *p*=0.682). Mothers were less likely to show aggression (table 1). We found no evidence that female body size (Wald_1_=0.04, *p*=0.850) or body weight (Wald_1_=0.13, *p*=0.718) was associated with aggression and no evidence that, within normal-horned females, horn size was associated with aggression (Wald_1_=0.13, *p*=0.715). Horn type remained significant when morphometric measures were included within the model (Wald_2_=4.68, *p*=0.035).

The occurrence of aggression depended upon the surrounding group structure (table 1). A high proportion of mothers in a group was associated with lower aggression (figure 2*a*). The higher the local density, the more likely an aggressive encounter (figure 2*b*). There was also significant interaction between density and proportion of mothers (table 1). Both results were not dependent upon an outlying high density, high-aggression group; removing the observation did not alter their significance when the model was rerun.

### (b) Aggressive interactions

Females were more likely to show aggression towards females who were younger, with positive coefficients for relative age (table 2). Independent of this association, females were more likely to show aggression towards females who displayed a horn phenotype associated with less weaponry than their own (table 2). We found no evidence that female body size (Wald_1_=0.12, *p*=0.720) or body weight (Wald_1_=1.50, *p*=0.220) influenced the outcome of aggression.

## 4. Discussion

We have shown that female horn development has a direct function in intrasexual aggressive competition for resources within a polygynous mammalian species. Females displaced individuals who displayed a horn phenotype associated with less weaponry than their own. This relationship was independent of the association between female aggression and age, with older females more likely to initiate aggression and displace younger ones. Males and females produce horns of different shapes and it has been hypothesized that the broad shape of male horns has evolved to withstand head-on clashes whereas female horns have evolved as spikes for displacing individuals (Lincoln 1994). Our results support the theory that the form of female horns observed in ungulates is a result of their function. They also indicate that female intrasexual aggression is context dependent. Aggressive interactions increased when local density was high, and are thus related to resource availability (Thouless & Guinness 1986; Festa-Bianchet 1991). Intrasexual aggression also decreased over the lambing period, with females distancing themselves from others to facilitate offspring protection.

However, it is unclear if an advantage in competition over resources conveys long-term benefits. In the short term, grass growth ceases over winter resulting in high mortality rates in late winter (Clutton-Brock & Pemberton 2004). Lambing follows this period; grass growth begins again in April, but lactation is costly and neonatal death is commonplace (Clutton-Brock & Pemberton 2004). Resource demand exceeds availability during this period and we predict that normal-horned females are better able to provision their offspring owing to their ability to gain more resources. Previous results from this system demonstrated that normal-horned females do not produce more offspring over their lifetime when compared with the other horn types (Robinson *et al.* 2006). An analysis of offspring fitness in relation to mother's horn type will reveal any benefits to an advantage in resource competition.

Genetic effects, testosterone levels and early developmental conditions may also influence aggressive behaviour and this may not be the only context in which females display aggression (e.g. Bro-Jørgensen 2002). Lower sample sizes may have resulted in reduced power to detect any effect of body or horn size, and although it is clear that age and horn phenotype directly influence female intrasexual interactions, other factors may be influential. However, it is clear that in studies of sexual selection, we must consider that a phenotype under sexual selection in males may also be under selection in females.

## Figures and Tables

**Figure 1:**
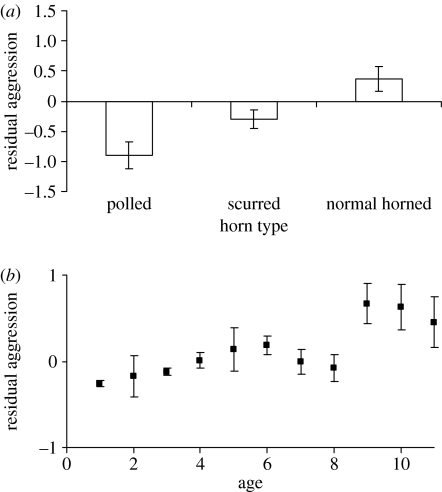
Probability of initiating aggression with (*a*) horn type and (*b*) age. Error bars represent standard error of the mean. *n*=862 Observations on 185 individuals.

**Figure 2:**
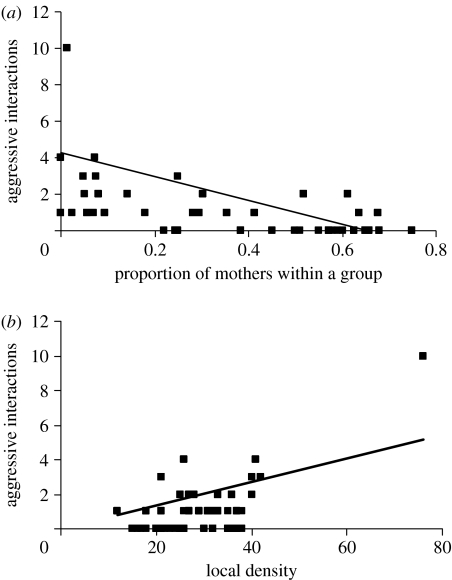
Number of aggressive interactions within a group with (*a*) proportion of mothers and (*b*) local density. *n*=50 Observation periods.

**Table 1:** Probability of aggression in all females observed. (Generalized linear mixed model with binomial error structure, showing only significant effects. Non-significant variables included body weight, body size and normal-horned female horn size. The random effect of individual accounted for repeated observations on females. *n*=862 Observations on 185 individuals.)

variable	estimate (s.e.)	d.f.	Wald statistic	*p*
*fixed effects*				
age	0.321 (0.055)	1	33.92	<0.001
horn type		2	28.59	<0.001
polled	−1.648 (0.774)			
scurred	0.000			
normal horned	1.152 (0.543)			
reproductive status		1	37.29	<0.001
without lamb	0.000			
with lamb	−2.628 (0.430)			
local density	0.017 (0.007)	1	6.14	0.014
proportion of mothers	−1.402 (0.506)	1	7.66	0.006
local density×proportion of mothers	−0.156 (0.062)	1	6.36	0.012

	variance (s.e.)			
*random effects*				
individual	4.706 (0.934)			
residual	4.210 (0.225)			

**Table 2:** Effects of relative age and relative horn type on the outcome of aggressive interactions in females. (Generalized linear mixed model with binomial error structure. The random effect of individual accounted for repeated interactions of individuals. *n*=51 Aggressive interaction involving 39 individuals.)

variable	estimate (s.e.)	d.f.	Wald statistic	*p*
*fixed effects*				
relative age	0.732 (0.201)	1	10.73	0.001
relative horn type	1.142 (0.576)	1	7.98	0.008
*random effects*				
individual	4.663 (3.202)			
residual	5.786 (1.365)			
